# Accelerated carbonation test data for concretes with varying slag content

**DOI:** 10.1016/j.dib.2026.112784

**Published:** 2026-04-16

**Authors:** Jukka Haavisto, Riku Paavilainen, Heikki Alho, Jukka Lahdensivu, Miguel Ferreira, Anssi Laaksonen

**Affiliations:** a Faculty of Built Environment – Concrete and Bridge Structures, Tampere University, Tampere, Finland; b Faculty of Built Environment – Renovation and Service Life Engineering of Structures, Tampere University, Tampere, Finland; c Structural Materials, VTT Technical Research Centre of Finland, Espoo, Finland

**Keywords:** Air-entrained, Cement content, Durability, GGBS, Low-carbon, w/b ratio

## Abstract

This paper presents a dataset of accelerated carbonation test results for 48 concrete mixes with varying binder compositions, water-to-binder ratios, and air-entrainment levels. The dataset includes raw and processed data on material properties, exposure conditions, carbonation depth measurements, and derived carbonation rates. Concrete mixes were prepared using four cement types with different proportions of ground granulated blast-furnace slag (GGBS), ranging from 0% to 72%. Each mix was tested in both air-entrained and non-air-entrained versions. Accelerated carbonation was performed in controlled chambers at approximately 3% CO₂ concentration. Carbonation depth was measured at multiple intervals up to 84 days using phenolphthalein indicator. Additional data cover fresh and hardened concrete properties. The dataset is openly available in Zenodo and can be reused for benchmarking mix designs, validating service-life models, and comparing accelerated and natural carbonation performance.

**Table:** Specifications table.

Subject	Engineering & Materials science
Specific subject area	Accelerated carbonation data of concrete
Type of data	Table (.xlsx format) and Image (.jpg format)Data format: Raw, Analyzed and Processed
Data collection	1. The material properties of the tested concretes were determined from the same batch that was used for the durability test specimens. The grading curve of aggregates was determined before mixing, and the binder properties were collected from the manufacturer’s batch reports. 2. Raw data on the exposure conditions (CO₂, temperature, RH) of the carbonation specimens were collected from sensors in the same location as the specimens. 3. Carbonation depth was measured on phenolphthalein-treated surfaces using a digital calliper (precision ±0.01 mm) and photographed. Average values per specimen and time point were calculated, and the carbonation rate was derived from a zero-intercept linear fit to the square root of exposure time.
Data source location	Durability test data and material property data for hardened concrete:Hervanta, Tampere, Finland, Latitude 61.450795° and Longitude 23.861168°Fresh concrete measurement data:Kauppi, Tampere, Finland, Latitude 61.504503° and Longitude 23.807576°Binder property data:Pargas, Finland, Latitude 60.285215° and Longitude 22.290729°
Data accessibility	Repository name: ZenodoData identification number: https://doi.org/10.5281/zenodo.19409803Direct URL to data: https://zenodo.org/records/19409803
Related research article	None

## 1 Value of the Data

• The dataset provides valuable insights into the effects of ground granulated blast furnace slag (GGBS) content, water-to-binder ratio, binder content, air entrainment and differences between the formwork surface and the cast surface on concrete carbonation. The dataset includes multiple concrete types tested using the same procedures and equipment, ensuring direct comparability of the results. The carbonation results can be utilized in service-life design to assess the influence of these parameters.
• Concrete producers can use the data when designing concrete mixes for specific carbonation exposure classes. The data is also useful for comparison with producers’ own test results, as accelerated carbonation testing is often selected in production-related laboratory testing due to its practical efficiency.
• Researchers can use the data when planning carbonation experiments and as a reference for their own measurement results. The dataset also supports the development and validation of carbonation models for slag-based concretes, which is important for designing durable concrete structures. The research topic is highly relevant, as service-life design is moving towards performance-based approaches and the use of low-carbon concretes is increasing.
• For concretes with high GGBS content, the dataset enables evaluation of the significance of curing duration under accelerated carbonation exposure. Concrete producers can use this information when selecting the reference age for their concrete mixes.
• In addition to carbonation results, the data can be used more broadly in concrete mix design and as background information to assess the influence of different parameters on compressive strength and density.
• The dataset is openly available and permanently accessible. This ensures transparency, reproducibility, and ease of reuse by all users.

## 2 Background

Cement production is estimated to contribute approximately 8% of global CO_2_ emissions [1]. Replacing Portland cement with supplementary cementitious materials, such as ground granulated blast-furnace slag (GGBS), can significantly reduce the carbon footprint of concrete [2,3]. However, the choice of binder has a considerable impact on concrete properties, particularly regarding the long-term durability of reinforced concrete structures [4,5].

Carbonation is one of the primary mechanisms that can reduce the service life of reinforced concrete by promoting reinforcement corrosion [6]. Reliable design with slag-based binders requires experimental data on how varying slag levels and concrete qualities influence carbonation. While carbonation rates for slag-based concretes have been published in several studies, data covering a broader range of mix designs, especially air-entrained concretes, remain limited.

In this dataset, carbonation was accelerated using an elevated CO₂ concentration. This enables faster and more cost-effective data generation than natural exposure, making it suitable for practical mix development and benchmarking [7]. In future work, selected concretes from this dataset will be exposed to natural carbonation conditions to support the calibration of different test methods.

## 3 Data Description

This article describes the dataset available in the linked Zenodo repository [8]. This dataset contains the results of accelerated carbonation tests for 48 different concrete mixes, as well as the general properties of these concretes.

A total of 216 concrete slabs were included in the accelerated carbonation testing program. For concretes made with CEM I or CEM II binders, three slabs were used per mix, all of which were water‑cured for 28 days. For concretes made with CEM III binders, six slabs were used per mix: three were water‑cured for 28 days, and three for 91 days. Thus, each curing condition consisted of three parallel slabs. Each slab contains two exposed surfaces relevant to carbonation testing: the cast surface and the mould surface. These were analysed as separate measurement series.

Measurements were performed at five exposure times. The first of these was taken at 0 days, prior to accelerated carbonation exposure, and was measured on one slab per series only. The initial carbonation depths were negligible, indicating no measurable carbonation prior to exposure, and these values are not included in the dataset. All subsequent measurements were performed on the three replicate slabs belonging to each series.

At each measurement time, carbonation depth was recorded at six predefined points on the cast surface and six corresponding points on the mould surface. An exception occurred for 23 of the earliest cast mixes, for which the first measurement at 7–13 days included three measurement points per surface instead of six. Depending on whether three or six measurements were taken at the first exposure time, each dataset corresponds to one concrete mix, one curing condition, and one surface contains either 63 or 72 individual carbonation depth values distributed over four exposure times.

Carbonation rates were calculated separately for each surface by fitting a zero‑intercept linear model between the mean carbonation depths at the four exposure times after the start of accelerated carbonation and the square root of exposure time.

The dataset consists of six summary tables presenting the compiled data for all the investigated concretes. These Excel files are named “TABLE_TITLE.xlsx”, where “TABLE” refers to the labelling (A–F), and “TITLE” to the table description, as shown in Table 1. Additionally, a separate zip archive is provided for each concrete mix, containing the image-based data from the carbonation-depth measurements and the slump test.

**Table 1:** Content of the related dataset (Excel files and their sheet structure) [8].

Table no.	Sheet no.	Title of the Table/Sheet	Content of the Sheet
**A**		**General properties**	
	00	- Cement properties	• Relative proportions of Portland clinker, limestone, and GGBS in the binder (%) • Compressive strength of the binder at 2, 7, and 28 days (MPa) • Specific surface area of the binder measured by Blaine method (m²/kg)
	01	- Aggregate grading	• Passing percentages of aggregate fractions for sieve sizes 0.063, 0.125, 0.25, 0.5, 1, 2, 4, 8, 16, 32, and 63 mm (%)
	02	- Concrete properties	• Binder type used and actual water-to-binder ratio • Binder, water, and aggregate contents in concrete (kg/m³), corrected for measured air content • Amounts of plasticizing and air-entraining admixtures relative to binder content (%) • Fresh concrete properties: slump (mm), air content (%), density (kg/m³), and temperature (°C) • Mean and standard deviation of compressive strength at 28 and 91 days (MPa) and density (kg/m³) of hardened concrete • Mean and standard deviation of hardened concrete total air void content (dm³/m³), vacuum saturation content (%), and capillary saturation content (%)
**B**	00 01	**Exposure calendar** - 28d cured specimens - 91d cured specimens	• Dates recorded for each concrete mix include the following stages: • Casting • Formwork removal and start of curing • End of curing and start of drying period • End of drying period and start of accelerated CO_2_ exposure • First carbonation depth measurement after 7–13 days of exposure • Second carbonation depth measurement after 21–29 days of exposure • Third carbonation depth measurement after 54–63 days of exposure • Fourth carbonation depth measurement after 81–84 days of exposure
**C**		**Drying conditions of carbonation specimens**	• Relative humidity (%) and temperature (°C) of the storage room during the drying period
**D**		**Raw data of chamber conditions**	
	00	- T and RH in chambers	• Relative humidity (%) and temperature (°C) in each of the three chambers during the accelerated CO_2_ exposure
	01	- CO_2_ in chambers	• CO_2_ concentration (%) in each of the three chambers during the accelerated CO_2_ exposure
**E**	00 01 02 03	**Carbonation depth measurements** - Carbonation depth 7–13d - Carbonation depth 21–29d - Carbonation depth 54–63d - Carbonation depth 81–84d	• Individual carbonation depth measurements (mm) from both cast and mould surfaces of each slab at four exposure times, including the mean value (mm) for each set of six measurements • Thickness of the test slab (mm)
**F**	00 01 02 03	**Carbonation rates** - Mould surface, 28d cured - Cast surface, 28d cured - Mould surface, 91d cured - Cast surface, 91d cured	• Carbonation rate (mm/d^0.5^) calculated as a zero-intercept linear fit of carbonation depth measurements at four exposure times for each specimen, including the mean and standard deviation for three parallel specimens • The coefficient of determination (R^2^) of the fitted model

**Fig. 1:**
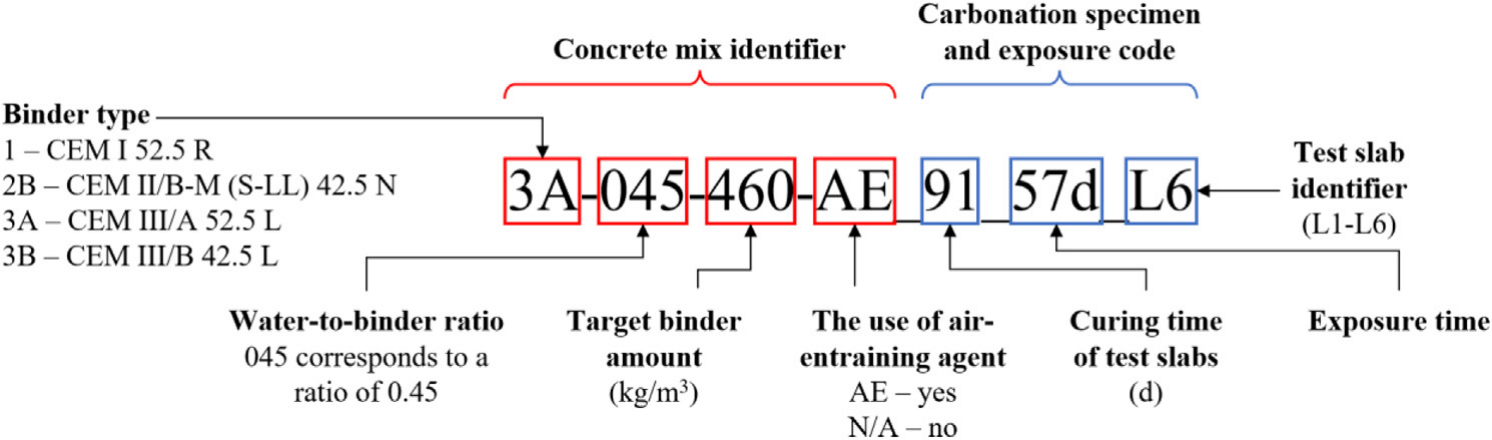
Naming of concrete mixes and accelerated carbonation images.

The studied concrete mixes are numbered from 1 to 48. In addition, they are coded in the data tables and zip archives in a way that the mix identifier reflects the variables modified in the mix design. In the carbonation image file names, the concrete identifier is followed by information on the specimen curing duration, the exposure time under accelerated carbonation, and a specimen-specific code, as shown in Fig. 1.

The carbonation depth measurement points are labelled according to the scheme shown in Fig. 6. The dataset also clearly distinguishes between measurements taken on the mould surface and on the cast surface of each test slab. Each image includes a millimetre scale, allowing the location of each measurement point to be traced back to the corresponding positions shown in Fig. 6.

## 4 Experimental Design, Materials and Methods

### 4.1 Materials

Accelerated carbonation tests were conducted on 48 different concrete mixes using four distinct types of cement as detailed in [9]. The compositions and key properties of the cement batches presented in Table 2 are derived from the cement supplier, Finnsementti Oy. The cement types exhibit significant variation in their GGBS content.

**Table 2:** Composition and properties of binders.

	CEM I 52.5 R	CEM II/B-M (S-LL) 42.5 N	CEM III/A 52.5 L	CEM III/B 42.5 L - LH/SR
*Composition (%)*				
GGBS	0	16	39	72
Limestone	3	8	5	2
Clinker (incl. sulphates)	97	76	56	26
*Compressive strength (MPa*)				
2 days	45.1	27.6	24.1	13.8
7 days	54.0	40.2	46.2	39.8
28 days	63.5	52.6	65.3	54.1
Blaine surface (m^2^/kg)	579	442	534	542

**Fig. 2:**
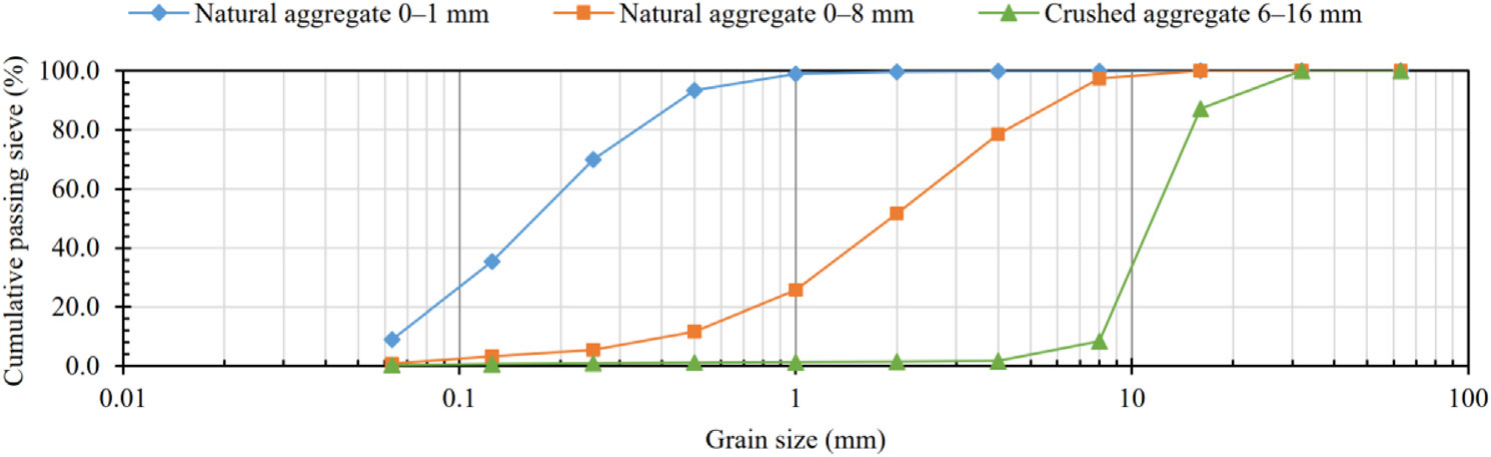
Grading curve of aggregates.

The aggregate for the concrete mixture was composed of three distinct fractions, the grain sizes of which are illustrated in Fig. 2. The moisture content of these aggregates, as determined by oven drying on each casting day, was taken into account in the proportioning of the concrete mix. The concrete mixtures also included water and a superplasticizer (Sika Viscoflow® MR-1), while air-entrained concrete mixtures contained an air-entraining agent (Sika AirPRO FI). The air-entraining agent was introduced as a 5% aqueous solution, prepared by diluting the required amount of admixture with the mixing water to ensure uniform distribution within the concrete.

The concrete mix designs were intentionally varied in terms of the type of cement utilized, the water-to-binder ratio (ranging from 0.39 to 0.60), and the target binder content (310 kg/m^3^ and 460 kg/m^3^). Each concrete mix was produced in both air-entrained and non-air-entrained versions.

The mix proportions for all concretes are presented in Table 3. The moisture content of the aggregates was measured on each casting day. In the table, the water content was adjusted for the free water carried by the aggregates, while the aggregate quantities account only for the absorbed moisture. For air-entrained concretes, the target air content was set at 5%. However, in most cases, the actual air content was significantly lower. Consequently, the mix proportions presented in Table 3 have been adjusted to align with the measured air content of each concrete mixture.

**Table 3:** Achieved concrete mix proportions.

No	Mix	Binder type	W/B	Binder (kg/m^3^)	Water (kg/m^3^)	Natural 0/1 (kg/m^3^)	Natural 0/8 (kg/m^3^)	Crushed 6/16 (kg/m^3^)	SP %*	AE %*
1	1–040–460	CEM I 52.5 R	0.40	468	187	99	957	657	2.50	-
2	1–040–460-AE		0.40	463	185	98	946	649	2.20	0.040
3	1–045–460		0.45	465	209	87	926	629	1.30	-
4	1–045–460-AE		0.45	453	204	85	902	613	1.20	0.040
5	1–050–460		0.50	466	234	73	900	605	0.40	-
6	1–050–460-AE		0.50	460	231	73	890	598	0.20	0.038
7	1–050–310		0.50	313	157	222	963	746	3.16	-
8	1–050–310-AE		0.50	309	156	220	952	737	2.90	0.040
9	1–055–310		0.55	313	172	216	948	732	1.90	-
10	1–055–310-AE		0.55	310	170	214	939	725	2.20	0.038
11	1–060–310		0.60	313	187	207	931	716	1.30	-
12	1–060–310-AE		0.60	309	184	204	918	706	1.50	0.032
13	2B-040–460	CEM II/B-M (S-LL) 42.5 N	0.40	469	188	99	959	658	1.40	-
14	2B–040–460-AE		0.40	465	186	98	949	652	1.18	0.034
15	2B-045–460		0.45	466	210	87	927	630	0.53	-
16	2B–045–460-AE		0.45	456	205	85	909	617	0.50	0.032
17	2B-050–460		0.50	466	234	73	900	605	0.20	-
18	2B–050–460-AE		0.50	460	231	73	889	598	0.20	0.030
19	2B-050–310		0.50	313	157	222	963	746	2.20	-
20	2B–050–310-AE		0.50	310	156	220	953	738	1.90	0.028
21	2B-055–310		0.55	313	172	216	947	731	1.55	-
22	2B–055–310-AE		0.55	303	167	209	918	709	0.90	0.028
23	2B-060–310		0.60	313	187	207	930	715	0.90	-
24	2B–060–310-AE		0.60	307	183	203	913	702	0.70	0.024
25	3A-040–460	CEM III/A 52.5 L	0.40	468	187	99	957	657	1.05	-
26	3A–040–460-AE		0.40	454	181	96	926	636	1.26	0.040
27	3A-045–460		0.45	468	210	87	931	632	0.70	-
28	3A–045–460-AE		0.45	456	205	85	908	616	0.78	0.038
29	3A-050–460		0.50	466	234	73	900	605	0.20	-
30	3A–050–460-AE		0.50	453	227	71	875	589	0.20	0.038
31	3A-050–310		0.50	311	157	221	957	741	1.56	-
32	3A–050–310-AE		0.50	308	155	218	946	733	1.70	0.032
33	3A-055–310		0.55	310	170	214	939	725	1.13	-
34	3A–055–310-AE		0.55	300	165	207	909	702	1.10	0.032
35	3A-060–310		0.60	310	185	205	921	709	0.70	-
36	3A–060–310-AE		0.60	302	180	199	898	691	0.50	0.032
37	3B-040–460	CEM III/B 42.5 L - LH/SR	0.39	470	185	99	962	659	1.00	-
38	3B–040–460-AE		0.39	441	173	93	904	618	0.80	0.032
39	3B-045–460		0.45	467	210	87	929	631	0.45	-
40	3B–045–460-AE		0.45	456	205	85	908	616	0.50	0.028
41	3B-050–460		0.50	467	234	74	902	607	0.20	-
42	3B–050–460-AE		0.50	464	233	73	897	603	0.20	0.028
43	3B-050–310		0.49	313	155	222	965	746	1.20	-
44	3B–050–310-AE		0.49	307	151	218	949	732	1.00	0.028
45	3B-055–310		0.55	310	171	214	940	726	0.60	-
46	3B–055–310-AE		0.55	309	170	214	937	724	1.30	0.024
47	3B-060–310		0.59	309	182	204	921	707	0.60	-
48	3B–060–310-AE		0.59	304	178	204	904	695	0.40	0.024

⁎ Percentage relative to the amount of binder.

### 4.2 Casting of specimens

The batch size ranged from 90 to 100 liters, contingent upon the specific mixture. The mixture was prepared using a pan mixer under controlled laboratory conditions. The fresh concrete properties, including workability, air content, and density, were determined for each batch according to EN 12350–2 [10], EN 12350–7 [11], and EN 12350–6 [12], respectively (see Table 4). While a superplasticizer was included, the concretes still demonstrated an unexpected range of workability. Photographs were taken from the slump test on each concrete. These images are stored and shared through the Zenodo database.

**Table 4:** Properties of the studied mixes.

No	Mix	Slump (mm)	Air content (%)	Density fresh (kg/m^3^)	Temperature	Density, (kg/m^3^)	*f*_cm_, 28d (MPa)	*f*_cm_, 91d (MPa)
1	1–050–310	70	0.7	2 447	22.0	2 450	63.2	70.7
2	1–050–310-AE	90	1.9	2 418	21.7	2 437	60.6	68.1
3	1–055–310	80	0.7	2 455	19.9	2 427	58.4	63.8
4	1–055–310-AE	70	1.7	2 428	21.4	2 403	55.3	59.8
5	1–060–310	140	0.6	2 424	20.5	2 420	50.6	55.8
6	1–060–310-AE	100	2.1	2 387	21.1	2 388	51.7	54.4
7	1–040–460	130	0.9	2 431	23.4	2 411	68.7	79.3
8	1–040–460-AE	170	2.1	2 394	20.4	2 381	62.9	72.1
9	1–045–460	120	1.7	2 388	21.5	2 393	63.1	72.6
10	1–045–460-AE	170	4.3	2 305	20.7	2 305	49.3	56.6
11	1–050–460	90	1.5	2 366	22.1	2 373	55.0	60.9
12	1–050–460-AE	100	2.7	2 326	21.5	2 324	45.9	53.9
13	2B-050–310	40	0.7	2 450	23.8	2 435	57.1	66.8
14	2B–050–310-AE	50	1.8	2 412	24.1	2 436	56.1	65.4
15	2B-055–310	80	0.8	2 429	23.2	2 415	51.7	61.1
16	2B–055–310-AE	80	4.0	2 332	21.3	2 372	41.9	49.1
17	2B-060–310	60	0.7	2 416	23.2	2 401	45.4	53.5
18	2B–060–310-AE	160	2.7	2 369	21.3	2 329	33.0	41.7
19	2B-040–460	100	0.7	2 441	24.7	2 410	60.6	71.1
20	2B–040–460-AE	160	1.7	2 392	23.8	2 381	58.0	67.1
21	2B-045–460	110	1.5	2 370	23.0	2 367	51.7	59.5
22	2B–045–460-AE	170	3.6	2 323	21.6	2 252	40.3	46.6
23	2B-050–460	170	1.5	2 342	23.1	2 341	45.5	53.7
24	2B–050–460-AE	180	2.8	2 307	21.7	2 285	39.8	47.2
25	3A-050–310	70	1.3	2 436	21.6	2 429	68.2	75.3
26	3A–050–310-AE	160	2.5	2 402	20.8	2 400	62.1	70.4
27	3A-055–310	150	1.7	2 392	20.7	2 415	61.7	69.1
28	3A–055–310-AE	220	5.0	2 317	20.8	2 317	43.4	51.6
29	3A-060–310	100	1.7	2 392	21.3	2 404	55.3	63.4
30	3A–060–310-AE	180	4.3	2 304	20.2	2 324	44.0	49.0
31	3A-040–460	140	0.9	2 419	22.4	2 410	78.1	84.7
32	3A–040–460-AE	210	4.2	2 310	20.9	2 345	67.8	73.4
33	3A-045–460	140	1.1	2 387	21.8	2 366	67.2	73.0
34	3A–045–460-AE	150	3.7	2 331	20.8	2 329	59.4	65.3
35	3A-050–460	110	1.5	2 341	21.5	2 332	55.4	62.7
36	3A–050–460-AE	170	4.4	2 259	19.4	2 276	47.8	55.3
37	3B-050–310	220	0.7	2 434	21.0	2 415	54.2	59.7
38	3B–050–310-AE	220	2.6	2 374	21.0	2 379	50.4	55.1
39	3B-055–310	40	1.6	2 402	22.4	2 392	48.5	53.0
40	3B–055–310-AE	220	1.9	2 393	16.8	2 362	45.0	50.5
41	3B-060–310	210	2.0	2 349	21.0	2 367	43.3	46.4
42	3B–060–310-AE	170	3.7	2 317	20.8	2 335	39.2	41.6
43	3B-040–460	-	0.6	2 367	21.3	2 400	64.4	69.6
44	3B–040–460-AE	-	7.0	2 236	21.1	2 279	51.3	54.4
45	3B-045–460	230	1.3	2 347	22.0	2 355	56.7	63.2
46	3B–045–460-AE	220	3.7	2 306	20.7	2 298	52.7	57.8
47	3B-050–460	200	1.3	2 322	21.7	2 327	49.3	53.5
48	3B–050–460-AE	140	1.9	2 312	20.2	2 321	50.7	52.8

150 mm × 300 mm cylindrical specimens were cast from each concrete batches. After demoulding, the cylinders were subjected to water immersion, in accordance with EN 12390–2 [13]. The compressive strength of these specimens was determined at 28 and 91 days in accordance with EN 12390–3 [14]. The compressive strength values presented in Table 4 are the average of three replicate specimens. Prior to the strength testing, the density of the cylinders was determined in accordance with EN 12390–7 [15]. The density of the hardened concrete presented in Table 4 is thus the average of six specimens.

**Fig. 3:**
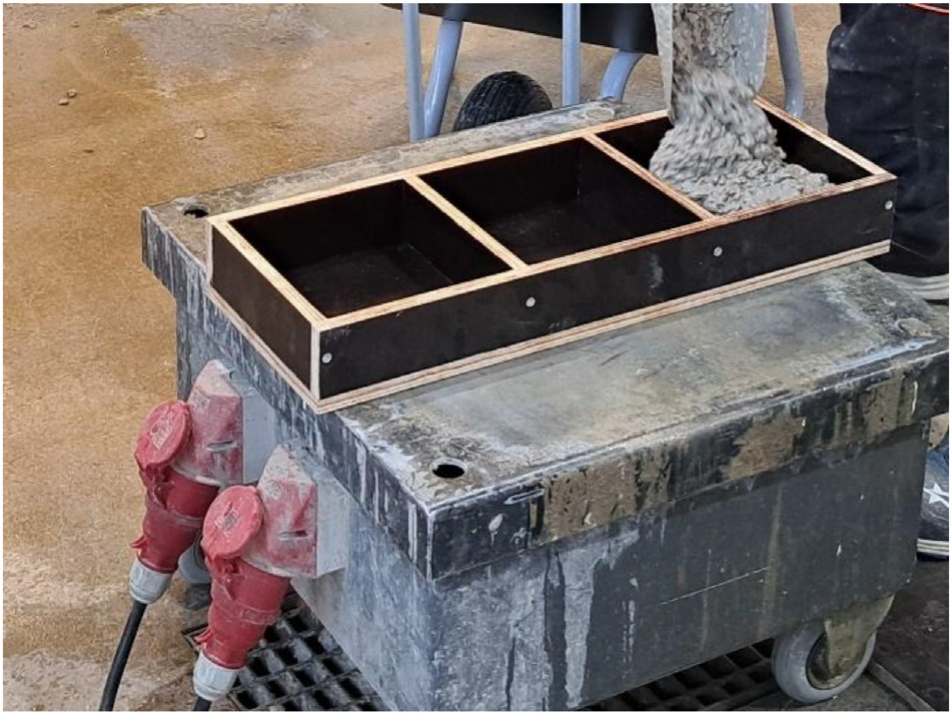
Casting of slabs for accelerated carbonation testing and vibrating table used.

For the carbonation tests, 200 mm × 200 mm concrete slabs were cast from each mix. The slab thickness was determined based on the water-to-binder (w/b) ratio used in the mix: 50 mm slabs were used for w/b ratios of 0.40 and 0.45, 60 mm for 0.50, and 70 mm for 0.55 and 0.60. All slabs were compacted using a vibrating table as shown in Fig. 3. The specimens were cast in film-faced plywood formwork, and the cast surface was finished by hand trowelling. After casting, the surface of each concrete slab was enveloped with a plastic film. For the accelerated carbonation tests, six replicate slabs were prepared from concretes made with CEM III-type cement, and three replicates for concretes with other binder types. From the same concrete batches, additional slabs and 100 mm diameter cylinders were cast for subsequent testing in other experimental phases.

### 4.3 Porosity tests

The porosity of the studied concretes was examined by determining the volumes of capillary pores and air-entrained pores using a procedure based on the standard SFS 4475 [16]. A notable deviation from the standard method was the utilization of vacuum saturation as opposed to pressurized water saturation, for the purpose of measuring the air-entrained pore volume. The primary aim of the testing program was to obtain comparable porosity results among the concretes under investigation.

The test specimens consisted of concrete discs with a diameter of 100 mm and a thickness of 40–70 mm, sawn from the upper portion of cylindrical concrete specimens originally cast to a height of 250–265 mm. Three discs were tested for each concrete mix. The specimens were demoulded one day after casting and subsequently stored underwater until the porosity tests commenced. At the time of testing, the concrete specimens were at least 56 days old.

Each specimen was dried in an oven at 105 °C for approximately three days to remove moisture from the pores. After drying, the specimen was equilibrated at ambient conditions (approximately 50% relative humidity) for 24 h. Subsequently, the mass of the solid phase was determined.

The specimen was subsequently placed in a water container with the water initially covering one-quarter of its height. The water level was raised by one every two hours until three-quarters of the specimen were submerged. After 24 h, the specimen was fully submerged and kept underwater for three days before re-weighing. The difference between this weight and the previous measurement was used to calculate the amount of water absorbed into the capillary pores.

In the final stage, the specimen underwent a second drying cycle, followed by vacuum saturation. This involved placing the specimen in an oven at 105 °C and then transferring it to a vacuum chamber filled with water. A vacuum of approximately one bar was applied for seven hours, after which the pump was turned off, leaving the specimen submerged for three to five days. After removal, the specimen was weighed again, and the increase in mass was used to determine the amount of water absorbed into the air-entrained pores.

The volume of each specimen was determined by measuring its weight in air and in water. The total volume of the specimen was used to determine the vacuum saturation content and the capillary saturation content.

### 4.4 Carbonation tests

#### 4.4.1 Test slab preparation and exposure conditions

Accelerated carbonation tests were carried out using a method based on EN 12390‑12 [17]. After casting, the surfaces of the concrete slabs were covered with a plastic film. The formwork was removed after 24 h, and the slabs were then transferred to water immersion. For each concrete mix, three slabs were kept immersed for 28 days. For concretes prepared with CEM III-type cements, an additional three slabs were immersed for 91 days.

Following water immersion, the slabs were allowed to dry under laboratory indoor conditions for 14 days. During this drying period, the relative humidity varied seasonally between 25 and 65%, with a temperature of about 20 °C.

**Fig. 4:**
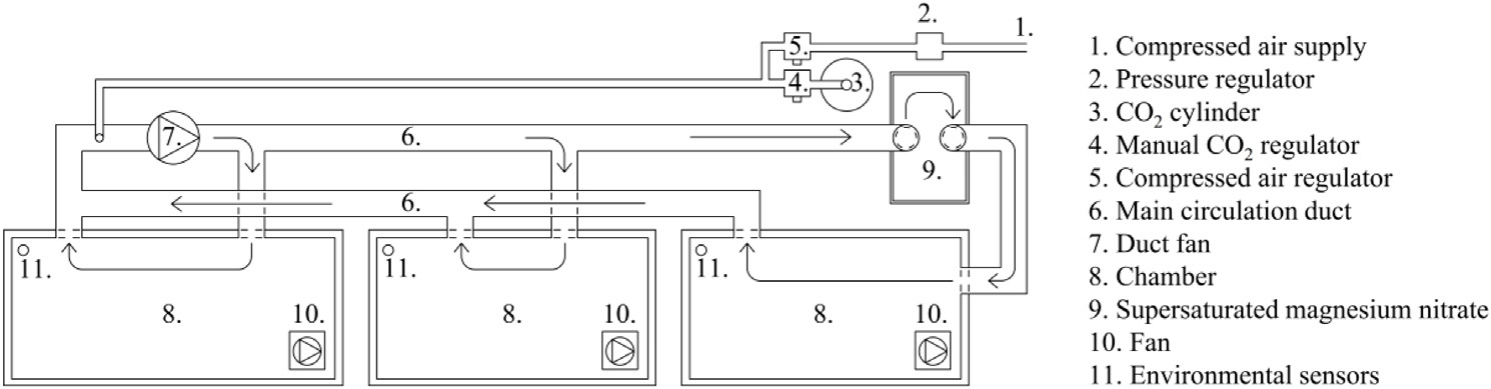
Schematic diagram of the system used for accelerated CO_2_ exposure.

After drying, the specimens were placed in accelerated CO₂ exposure chambers, schematically shown in Fig. 4. The system consisted of a CO₂ supply, a main circulation duct, a humidity control unit, three parallel chambers, and chamber-specific fans and sensors. CO₂ was introduced as a mixed gas with air to maintain a uniform concentration. The airflow rate in the circulation system exceeds 1 m³/min and is distributed across the three carbonation chambers. The rapid and uniform response of CO₂ concentration to control adjustments in all chambers indicates that air exchange was sufficient for homogeneous exposure conditions.

**Fig. 5:**
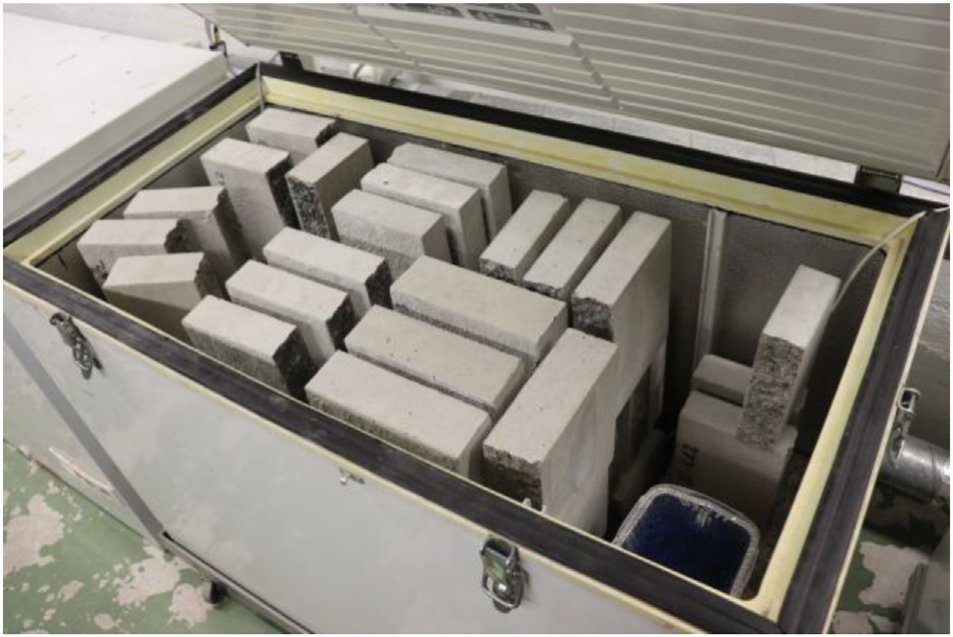
Storage of test slabs in accelerated carbonation chambers.

The slabs were arranged in three layers inside the chambers (Fig. 5) with sufficient spacing to ensure exposure of the measurement surfaces to the circulating gas mixture. The specimens were arranged as far apart as permitted by the available chamber space and the practical constraints of the test setup. The test slabs were arranged in the chambers according to their casting dates. This allowed rapid collection of specimens at measurement times and minimized the duration during which the chamber gas mixture was exposed to room air. To reduce potential variations in exposure conditions arising from the storage position, air was circulated using chamber‑specific fans. The environmental conditions for each chamber were measured separately to ensure uniform exposure.

Carbon dioxide concentration was measured using factory‑calibrated sensors (type INIR2‑CD100). Temperature and relative humidity were measured with sensors (type DT 172 CEM) with accuracies of ±2%RH and ±0.5 °C. Temperature and relative humidity were recorded every hour, and carbon dioxide concentration every 10 min.

All analyses were performed using raw time-series data extracted directly from the sensors. The raw datasets from each sensor were exported without any filtering, smoothing, or other preprocessing. After extraction, the datasets were only combined into a single Excel file for further analysis.

The target conditions in the chambers were 3% CO₂ concentration, 20 °C temperature, and 55% relative humidity. However, the relative humidity deviated from the target, particularly during the initial phase of testing period. Humidity control was initially achieved using a saturated magnesium nitrate solution, which stabilizes RH at about 53%. As the number of specimens increased, the moisture load in the chambers rose, and the solution could not maintain sufficiently low RH due to its high solubility. Additional moisture was introduced by the relatively high indoor humidity at the start of the experiment, as the compressed air for the gas mixture was sourced from indoor air. Consequently, silica gel was used for active moisture removal, enabling RH to be maintained at an average of 65%. As the concretes dried and indoor humidity decreased during winter, the chamber RH gradually dropped to an average of 55% in the latter half of the experiment period.

### 4.5 Carbonation depth measurements

Carbonation depths were determined for each specimen at four exposure intervals: 6–14 days, 21–29 days, 56–63 days, and 81–84 days. In addition, for each series, carbonation depth was measured on one slab prior to the start of the accelerated carbonation exposure to confirm that no measurable carbonation had occurred at the initial state.

For each measurement, the specimen was removed from the chamber and a strip approximately 20–30 mm thick was cut using a mechanical tile cutter. The remaining specimen was returned immediately to the chamber. The freshly cut surface was cleaned with compressed air and sprayed with a solution containing 1% phenolphthalein and 70% ethanol, applied evenly to avoid streaking. Phenolphthalein stains concrete dark purple at pH > 8–10, allowing the carbonation front to be identified. Each measurement was photographed, and the images were stored and shared via the Zenodo database [8].

**Fig. 6:**
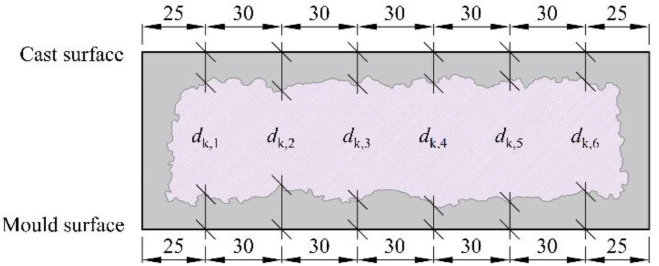
Locations of predefined measurement points for carbonation depth on test slabs.

Carbonation depth was measured at six predefined points on both the cast and formwork surfaces, which were analysed separately due to their differing surface characteristics. The measurement point locations are illustrated in Fig. 6. If a large aggregate or void was encountered at the measurement point, the depth was taken adjacent to its edge.

The practical measurement uncertainty of the carbonation depth readings was estimated to be approximately ±0.5 mm. This estimate reflects the combined effect of the digital calliper’s precision and the sharpness of the phenolphthalein colour transition. All measurements were performed by a single operator, which minimised variability associated with operator differences.

### 4.6 Carbonation rate determination

Carbonation rates were derived separately for the cast and mould surfaces of each slab. For each surface, the mean carbonation depths at the four exposure times after the start of accelerated carbonation were plotted against the square root of exposure duration, and a zero‑intercept linear model was fitted. This procedure follows the principle adopted in EN 12390‑12 [17], where the square‑root‑of‑time behaviour is assumed and the carbonation depth at the onset of exposure is zero. Initial measurements performed on slabs at 0 days confirmed that carbonation was negligible prior to accelerated CO_2_ exposure, supporting the application of a zero‑intercept approach. The resulting coefficients of determination (R²) indicate that the zero‑intercept linear fit performs well for most of the investigated concretes, with R² values typically exceeding 0.95. In concretes with the highest resistance to carbonation, R² values were occasionally lower, reflecting the fact that carbonation depths at one or more exposure times were very close to zero.

## Limitations

This dataset is limited to concretes with achieved w/b-ratios between 0.39 and 0.60, binder contents ranging from 300 to 470 kg/m³, and binders where GGBS is the only hydraulic additive used alongside Portland cement clinker.

In several air‑entrained mixtures, the air‑entrainment process did not perform as intended, leading to reduced air contents. As a result, these mixes may not correspond to frost-resistant concrete grades.

This dataset is restricted to carbonation progression in uncracked concrete, produced under laboratory conditions, and exposed to accelerated CO₂ environments. Carbonation under natural CO₂ concentrations, other long-term deterioration mechanisms, and the combined effects of multiple exposure conditions are not included.

The relative humidity in the chamber during accelerated carbonation testing showed seasonal variation and deviated from the target value of 55%, ranging approximately between 50% and 70%, particularly in the early stages of the exposure period.

The specimens may have retained moisture at the beginning of exposure despite the two-week drying period, which could have influenced carbonation progression. Moreover, the extent of drying achieved under the laboratory drying procedure may vary between specimens, and this variability could also affect the early stages of carbonation development.

Specimen thickness varied between mixes. Although thicker slabs initially contained a larger total amount of water, these mixes also had higher water-to-binder ratios and therefore higher capillary porosity. As a result, the overall influence of slab thickness on the drying rate is expected to be limited, and slab thickness is not considered to have had a meaningful effect on carbonation progression.The main sources of uncertainty in carbonation-depth measurements include the interpretation of the phenolphthalein colour-change boundary, local unevenness of the cut surface and the presence of coarse aggregate particles near measurement points. These uncertainties were mitigated by following the procedures of EN 12390-12 [17], performing six independent parallel measurements on each surface, and reporting all raw measurements to allow assessment of measurement scatter. In addition, all measurements were carried out by the same operator, reducing variation related to subjective interpretation.

The size of the test slabs differs from real structural elements. This may influence drying behaviour, segregation of concrete, and carbonation progression.

## Ethics Statement

The authors have read and follow the ethical requirements for publication in Data in Brief and confirming that the current work does not involve human subjects, animal experiments, or any data collected from social media platforms.

## CRediT authorship contribution statement

**Jukka Haavisto:** Data curation, Writing – original draft, Visualization, Project administration, Funding acquisition. **Riku Paavilainen:** Formal analysis, Investigation, Data curation, Writing – review & editing, Visualization. **Heikki Alho:** Investigation, Data curation, Writing – review & editing. **Jukka Lahdensivu:** Writing – review & editing, Supervision, Funding acquisition. **Miguel Ferreira:** Writing – review & editing, Supervision. **Anssi Laaksonen:** Writing – review & editing, Supervision, Funding acquisition.

## Data Availability

(Zenodo).Dataset from accelerated carbonation tests on concretes with varying slag content (Original data)
